# 2-Meth­oxy-4-(2-meth­oxy­phen­yl)-5,6,7,8,9,10-hexa­hydro­cyclo­octa­[*b*]pyridine-3-carbo­nitrile

**DOI:** 10.1107/S1600536814010332

**Published:** 2014-05-10

**Authors:** R. Vishnupriya, J. Suresh, S. Maharani, R. Ranjith Kumar, P. L. Nilantha Lakshman

**Affiliations:** a Department of Physics, The Madura College, Madurai 625 011, India; b Department of Organic Chemistry, School of Chemistry, Madurai Kamaraj University, Madurai 625 021, India; c Department of Food Science and Technology, University of Ruhuna, Mapalana, Kamburupitiya 81100, Sri Lanka

## Abstract

In the title compound, C_20_H_22_N_2_O_2_, the central pyridine ring forms a dihedral angle of 76.32 (8)° with the pseudo-axial benzene ring. The cyclo­octane ring adopts a twisted boat chair conformation. In the crystal, weak inter­molecular C—H⋯π inter­actions between inversion-related mol­ecules result in the formation of linear double chains along the *b*-axis direction.

## Related literature   

For the biological activities of substituted pyridine derivatives, see: Yao *et al.* (1994 ▶); Lohaus & Dittmar (1968 ▶). For a description of structure correlation, bond lengths and angles, see: Allen *et al.* (1987 ▶). For ring conformation parameters, see: Cremer & Pople (1975 ▶). The linearity of the cyano group seen in the title compound is typical of this class of 2-oxo­pyridine-3-carbo­nitrile compounds, see: Black *et al.* (1992 ▶); Hussain *et al.* (1996 ▶).
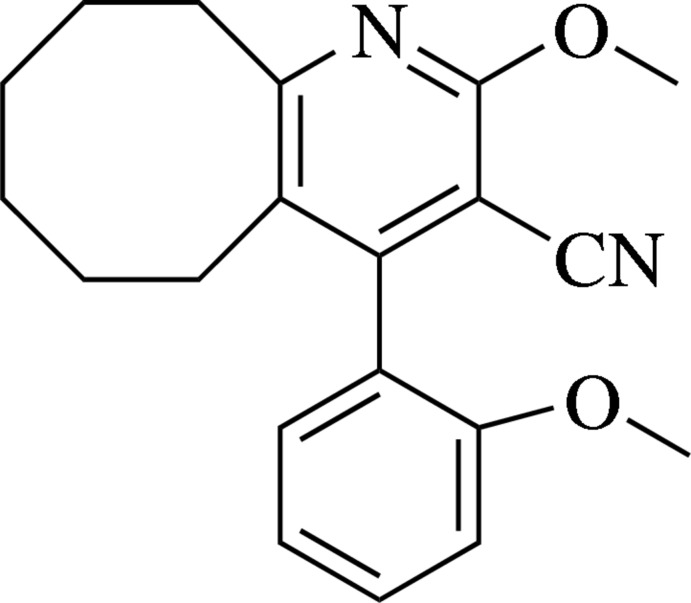


## Experimental   

### 

#### Crystal data  

C_20_H_22_N_2_O_2_
*M* *_r_* = 322.40
Monoclinic, 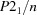

*a* = 11.1652 (10) Å
*b* = 11.4205 (9) Å
*c* = 14.8540 (11) Å
β = 111.763 (2)°
*V* = 1759.1 (2) Å^3^
*Z* = 4
Mo *K*α radiation
μ = 0.08 mm^−1^
*T* = 293 K
0.30 × 0.29 × 0.25 mm

#### Data collection  

Bruker Kappa APEXII diffractometer
Absorption correction: multi-scan (*SADABS*; Sheldrick, 1996 ▶) *T*_min_ = 0.977, *T*_max_ = 0.981
23618 measured reflections
3266 independent reflections
2251 reflections with *I* > 2σ(*I*)
*R*_int_ = 0.029

#### Refinement  

*R*[*F* ^2^ > 2σ(*F* ^2^)] = 0.050
*wR*(*F* ^2^) = 0.137
*S* = 1.05
3266 reflections
217 parameters
H-atom parameters constrained
Δρ_max_ = 0.24 e Å^−3^
Δρ_min_ = −0.29 e Å^−3^

### 

Data collection: *APEX2* (Bruker, 2004 ▶); cell refinement: *SAINT* (Bruker, 2004 ▶); data reduction: *SAINT*; program(s) used to solve structure: *SHELXS97* (Sheldrick, 2008 ▶); program(s) used to refine structure: *SHELXL97* (Sheldrick, 2008 ▶); molecular graphics: *PLATON* (Spek, 2009 ▶); software used to prepare material for publication: *SHELXL97*.

## Supplementary Material

Crystal structure: contains datablock(s) global, I. DOI: 10.1107/S1600536814010332/gg2137sup1.cif

Structure factors: contains datablock(s) I. DOI: 10.1107/S1600536814010332/gg2137Isup2.hkl

Supporting information file. DOI: 10.1107/S1600536814010332/gg2137Isup3.cml

CCDC reference: 1001458

Additional supporting information:  crystallographic information; 3D view; checkCIF report

## Figures and Tables

**Table 1:** Hydrogen-bond geometry (Å, °) *Cg*1 is the centroid of the pyridine ring.

*D*—H⋯*A*	*D*—H	H⋯*A*	*D*⋯*A*	*D*—H⋯*A*
C3—H3*A*⋯*Cg*1^i^	0.97	2.64	3.742 (2)	134

Symmetry code: (i) 
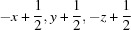
.

**Fig. 1.**
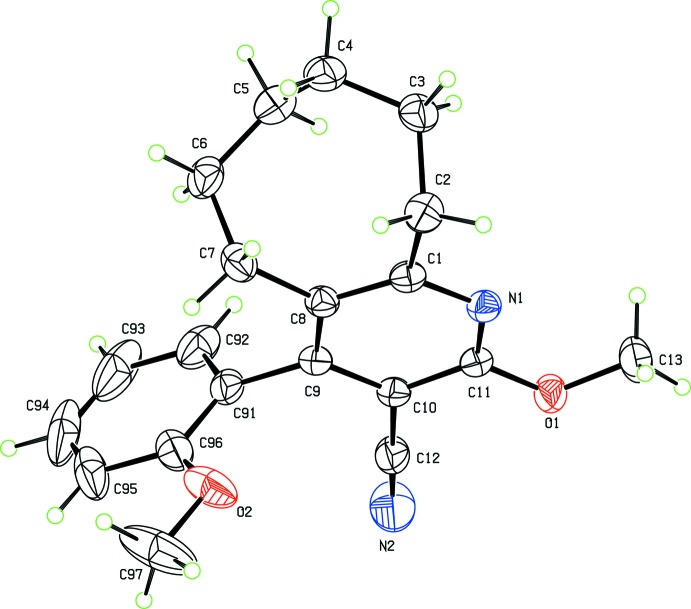
The molecular structure of compound showing 30% probability displacement ellipsoids and the atom-numbering scheme.

**Fig. 2.**
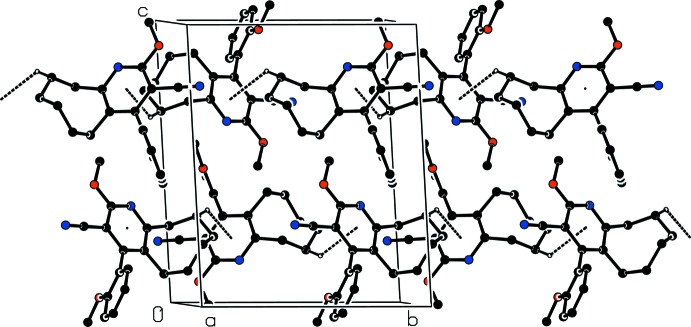
Partial packing view of the compound showing molecules interconnected through a C—H···π stacking interaction (dotted lines; symmetry code: (i) 1/2 - x, 1/2 + y, 1/2 - z)

## supplementary crystallographic information

### 1. Comment

Pyridine derivatives have a wide range of biological activities being used as fungicidal, antibacterial, antifungal, antimycotic (Lohaus *et al.*, 1968) and antidepressant agents, as well as thienopyridines being used as antithrombotic agents (Yao *et al.*, 1994) against platelet aggregation. The above observations prompted us to synthesize the title compound containing pyridine carbonitrile groups and substitued pyridine scaffolds to determine its crystal structure.

The molecular structure of the title compound is shown in Fig 1. The cyclooctane ring (C1–C8) adopts a twisted boat chair conformation as evidenced by the puckering parameters q_2_ = 1.1578 (8) Å, θ = 67.05 (2)°, φ = 103.05 (2)° (Cremer & Pople, 1975). The central pyridine component is planar, with a maximum deviation from the mean plane that of 0.0092 (1) Å for atom C10. The shortening of the C–N distances [1.354 (3) and 1.314 (3) Å] and the opening of the N1–C11–C10 angle [123.46 (2)°] may be attributed to the size of the substituent at C1. The sum of the C—N—C bond angle around N1 atom (365.0 (6)°) is implying a noticeable flattening of the trigonal pyramidal geometry about N1. The C10—C12 ≡N2 bond angle of 178.27 (1)° defining the linearity of the cyano group are typical of this group of 2-oxopyridine-3-carbonitrile compounds (Black *et al.*, 1992; Hussain *et al.*, 1996). The bond distances in the central pyridine ring range 1.314 (1) – 1.400 (2) Å suggest possible delocalization of the π electrons over the ring (Allen *et al.*, 1987). The dihedral angle between the pseudo-axial phenyl substituent and the plane of the pyridine ring is 76.32 (8)°. Due to conjugation, the bond length C11—O1 ═1.349 (4) Å is shorter than the bond length C13—O1 ═ 1.427 (2) Å.

The molecular structure features a weak intermolecular C—H···*Cg*1 interaction between inverse related molecules forming a linear double chain along *b* axis. [symmetry code: (i) 1/2 - *x*, 1/2 + *y*, 1/2 - *z*](Fig 2)(*Cg*1 is the centroid of the pyridine ring N1/C1/C8–C11).

### 2. Experimental

Preparation: A mixture of cyclooctanone (1 mmol), 2-methoxy benzaldehyde (1 mmol) and malononitrile (1 mmol) were taken in methanol (10 ml) to which lithium ethoxide (1 equiv) was added. The reaction mixture was heated under reflux for 2–3 h. After completion of the reaction (TLC), the reaction mixture was poured into crushed ice and extracted with ethyl acetate. The excess solvent was removed under vacuum and the residue was subjected to column chromatography using petroleum ether/ ethyl acetate mixture (95:5 *v*/*v*) as eluent to obtain pure product. Melting point:151–156 °C, Yield: 71%.

### 3. Refinement

H atoms were placed at calculated positions and allowed to ride on their carrier atoms with C—H = 0.93–0.98 Å and with *U*_iso_ = 1.2*U*_eq_(C, N) for N, CH_2_ and CH atoms and *U*_iso_ = 1.5*U*_eq_(C) for CH_3_ atoms.

### Crystal data

**Table:**

C_20_H_22_N_2_O_2_	*F*(000) = 688
*M**_r_* = 322.40	*D*_x_ = 1.217 Mg m^−^^3^
Monoclinic, *P*2_1_/*n*	Mo *K*α radiation, λ = 0.71073 Å
Hall symbol: -P 2yn	Cell parameters from 2000 reflections
*a* = 11.1652 (10) Å	θ = 2–26°
*b* = 11.4205 (9) Å	µ = 0.08 mm^−^^1^
*c* = 14.8540 (11) Å	*T* = 293 K
β = 111.763 (2)°	Block, colourless
*V* = 1759.1 (2) Å^3^	0.30 × 0.29 × 0.25 mm
*Z* = 4	

### Data collection

**Table:**

Bruker Kappa APEXII diffractometer	3266 independent reflections
Radiation source: fine-focus sealed tube	2251 reflections with *I* > 2σ(*I*)
Graphite monochromator	*R*_int_ = 0.029
Detector resolution: 0 pixels mm^-1^	θ_max_ = 25.5°, θ_min_ = 2.3°
ω and φ scans	*h* = −13→13
Absorption correction: multi-scan (*SADABS*; Sheldrick, 1996)	*k* = −13→13
*T*_min_ = 0.977, *T*_max_ = 0.981	*l* = −17→17
23618 measured reflections	

### Refinement

**Table:**

Refinement on *F*^2^	Primary atom site location: structure-invariant direct methods
Least-squares matrix: full	Secondary atom site location: difference Fourier map
*R*[*F*^2^ > 2σ(*F*^2^)] = 0.050	Hydrogen site location: inferred from neighbouring sites
*wR*(*F*^2^) = 0.137	H-atom parameters constrained
*S* = 1.05	*w* = 1/[σ^2^(*F*_o_^2^) + (0.0486*P*)^2^ + 0.8085*P*] where *P* = (*F*_o_^2^ + 2*F*_c_^2^)/3
3266 reflections	(Δ/σ)_max_ < 0.001
217 parameters	Δρ_max_ = 0.24 e Å^−^^3^
0 restraints	Δρ_min_ = −0.29 e Å^−^^3^

### Special details

**Table:**

Geometry. All e.s.d.'s (except the e.s.d. in the dihedral angle between two l.s. planes) are estimated using the full covariance matrix. The cell e.s.d.'s are taken into account individually in the estimation of e.s.d.'s in distances, angles and torsion angles; correlations between e.s.d.'s in cell parameters are only used when they are defined by crystal symmetry. An approximate (isotropic) treatment of cell e.s.d.'s is used for estimating e.s.d.'s involving l.s. planes.
Refinement. Refinement of *F*^2^ against ALL reflections. The weighted *R*-factor *wR* and goodness of fit *S* are based on *F*^2^, conventional *R*-factors *R* are based on *F*, with *F* set to zero for negative *F*^2^. The threshold expression of *F*^2^ > σ(*F*^2^) is used only for calculating *R*-factors(gt) *etc*. and is not relevant to the choice of reflections for refinement. *R*-factors based on *F*^2^ are statistically about twice as large as those based on *F*, and *R*- factors based on ALL data will be even larger.

### Fractional atomic coordinates and isotropic or equivalent isotropic displacement parameters (Å^2^)

**Table:**

	*x*	*y*	*z*	*U*_iso_*/*U*_eq_	
C1	0.13770 (18)	0.36565 (16)	0.23039 (14)	0.0433 (5)	
C2	0.2136 (2)	0.47203 (18)	0.22349 (17)	0.0564 (6)	
H2A	0.2578	0.4544	0.1798	0.068*	
H2B	0.2790	0.4871	0.2870	0.068*	
C3	0.1343 (2)	0.58356 (19)	0.18823 (16)	0.0601 (6)	
H3A	0.1858	0.6383	0.1679	0.072*	
H3B	0.0594	0.5639	0.1313	0.072*	
C4	0.0876 (2)	0.64636 (19)	0.25967 (17)	0.0629 (6)	
H4A	0.1615	0.6598	0.3190	0.075*	
H4B	0.0545	0.7224	0.2328	0.075*	
C5	−0.0158 (2)	0.5844 (2)	0.28588 (18)	0.0645 (6)	
H5A	−0.0783	0.6423	0.2879	0.077*	
H5B	−0.0604	0.5297	0.2343	0.077*	
C6	0.0304 (3)	0.5184 (2)	0.38079 (17)	0.0655 (6)	
H6A	−0.0430	0.4790	0.3873	0.079*	
H6B	0.0624	0.5746	0.4332	0.079*	
C7	0.1354 (2)	0.42796 (19)	0.39301 (15)	0.0574 (6)	
H7A	0.2147	0.4683	0.3998	0.069*	
H7B	0.1505	0.3844	0.4523	0.069*	
C8	0.10287 (18)	0.34305 (16)	0.30965 (13)	0.0424 (4)	
C9	0.03020 (18)	0.24227 (16)	0.30695 (13)	0.0407 (4)	
C10	−0.00175 (18)	0.16888 (16)	0.22689 (13)	0.0413 (4)	
C11	0.03687 (18)	0.20046 (16)	0.15077 (13)	0.0419 (5)	
C12	−0.0716 (2)	0.06187 (19)	0.22081 (15)	0.0559 (6)	
C13	0.0307 (3)	0.1604 (2)	−0.00811 (16)	0.0795 (8)	
H13A	−0.0003	0.1013	−0.0574	0.119*	
H13B	0.1223	0.1683	0.0108	0.119*	
H13C	−0.0102	0.2337	−0.0330	0.119*	
C91	−0.0182 (2)	0.21394 (17)	0.38544 (15)	0.0506 (5)	
C92	−0.1455 (3)	0.2350 (2)	0.3709 (2)	0.0728 (7)	
H92	−0.2006	0.2661	0.3124	0.087*	
C93	−0.1909 (4)	0.2101 (3)	0.4427 (4)	0.1232 (16)	
H93	−0.2770	0.2239	0.4327	0.148*	
C94	−0.1098 (7)	0.1649 (3)	0.5291 (4)	0.140 (2)	
H94	−0.1412	0.1487	0.5776	0.167*	
C95	0.0175 (5)	0.1433 (3)	0.5450 (2)	0.1061 (13)	
H95	0.0724	0.1130	0.6040	0.127*	
C96	0.0628 (3)	0.1673 (2)	0.47174 (16)	0.0682 (7)	
C97	0.2778 (4)	0.1043 (3)	0.5663 (2)	0.1477 (19)	
H97A	0.3596	0.0935	0.5599	0.222*	
H97B	0.2485	0.0308	0.5818	0.222*	
H97C	0.2871	0.1596	0.6171	0.222*	
N1	0.10398 (15)	0.29525 (13)	0.15152 (11)	0.0449 (4)	
N2	−0.1246 (3)	−0.0247 (2)	0.21553 (17)	0.0915 (8)	
O1	0.00127 (16)	0.12727 (12)	0.07402 (10)	0.0586 (4)	
O2	0.1868 (2)	0.14718 (16)	0.47771 (13)	0.0875 (6)	

### Atomic displacement parameters (Å^2^)

**Table:**

	*U*^11^	*U*^22^	*U*^33^	*U*^12^	*U*^13^	*U*^23^
C1	0.0415 (11)	0.0372 (10)	0.0509 (11)	−0.0009 (8)	0.0167 (9)	0.0006 (9)
C2	0.0571 (13)	0.0514 (13)	0.0674 (14)	−0.0139 (10)	0.0309 (11)	−0.0060 (11)
C3	0.0770 (16)	0.0461 (12)	0.0592 (13)	−0.0144 (11)	0.0276 (12)	0.0010 (10)
C4	0.0774 (16)	0.0416 (12)	0.0674 (15)	−0.0042 (11)	0.0244 (12)	−0.0003 (11)
C5	0.0678 (15)	0.0527 (13)	0.0741 (16)	0.0009 (11)	0.0275 (13)	−0.0095 (12)
C6	0.0912 (18)	0.0495 (13)	0.0666 (15)	−0.0145 (12)	0.0418 (13)	−0.0169 (11)
C7	0.0752 (15)	0.0499 (12)	0.0419 (11)	−0.0153 (11)	0.0155 (10)	−0.0025 (9)
C8	0.0453 (11)	0.0368 (10)	0.0417 (10)	−0.0017 (8)	0.0121 (9)	0.0003 (8)
C9	0.0440 (10)	0.0351 (9)	0.0429 (10)	0.0029 (8)	0.0159 (8)	0.0036 (8)
C10	0.0470 (11)	0.0329 (9)	0.0451 (10)	0.0001 (8)	0.0182 (9)	0.0026 (8)
C11	0.0489 (11)	0.0338 (10)	0.0431 (10)	0.0033 (8)	0.0172 (9)	−0.0002 (8)
C12	0.0764 (15)	0.0459 (12)	0.0517 (12)	−0.0099 (11)	0.0309 (11)	−0.0051 (10)
C13	0.137 (2)	0.0623 (15)	0.0528 (13)	−0.0205 (16)	0.0502 (15)	−0.0071 (12)
C91	0.0692 (14)	0.0376 (10)	0.0517 (12)	−0.0090 (10)	0.0300 (11)	−0.0072 (9)
C92	0.0771 (17)	0.0589 (15)	0.103 (2)	−0.0064 (13)	0.0573 (16)	−0.0143 (14)
C93	0.164 (4)	0.089 (2)	0.191 (4)	−0.038 (2)	0.151 (4)	−0.050 (3)
C94	0.277 (7)	0.083 (2)	0.139 (4)	−0.083 (3)	0.171 (5)	−0.056 (2)
C95	0.214 (4)	0.0631 (18)	0.0604 (16)	−0.058 (2)	0.074 (2)	−0.0173 (13)
C96	0.107 (2)	0.0470 (13)	0.0490 (13)	−0.0246 (13)	0.0276 (14)	−0.0059 (11)
C97	0.147 (3)	0.098 (2)	0.105 (3)	−0.044 (2)	−0.061 (2)	0.048 (2)
N1	0.0512 (10)	0.0376 (9)	0.0501 (9)	0.0003 (7)	0.0237 (8)	0.0013 (7)
N2	0.133 (2)	0.0638 (14)	0.0897 (16)	−0.0438 (14)	0.0552 (15)	−0.0150 (12)
O1	0.0903 (11)	0.0440 (8)	0.0488 (8)	−0.0127 (8)	0.0342 (8)	−0.0068 (7)
O2	0.0884 (14)	0.0793 (13)	0.0679 (11)	−0.0022 (10)	−0.0025 (10)	0.0277 (9)

### Geometric parameters (Å, º)

**Table:**

C1—N1	1.354 (2)	C10—C11	1.399 (3)
C1—C2	1.506 (3)	C10—C12	1.434 (3)
C1—C8	1.394 (3)	C11—N1	1.314 (2)
C2—C3	1.529 (3)	C11—O1	1.349 (2)
C2—H2A	0.9700	C12—N2	1.140 (3)
C2—H2B	0.9700	C13—O1	1.427 (3)
C3—C4	1.524 (3)	C13—H13A	0.9600
C3—H3A	0.9700	C13—H13B	0.9600
C3—H3B	0.9700	C13—H13C	0.9600
C4—C5	1.523 (3)	C91—C96	1.373 (3)
C4—H4A	0.9700	C91—C92	1.377 (3)
C4—H4B	0.9700	C92—C93	1.370 (4)
C5—C6	1.511 (3)	C92—H92	0.9300
C5—H5A	0.9700	C93—C94	1.368 (6)
C5—H5B	0.9700	C93—H93	0.9300
C6—C7	1.522 (3)	C94—C95	1.374 (6)
C6—H6A	0.9700	C94—H94	0.9300
C6—H6B	0.9700	C95—C96	1.387 (4)
C7—C8	1.507 (3)	C95—H95	0.9300
C7—H7A	0.9700	C96—O2	1.373 (3)
C7—H7B	0.9700	C97—O2	1.418 (3)
C8—C9	1.400 (3)	C97—H97A	0.9600
C9—C10	1.389 (3)	C97—H97B	0.9600
C9—C91	1.491 (3)	C97—H97C	0.9600
			
N1—C1—C8	123.06 (17)	C10—C9—C91	119.08 (16)
N1—C1—C2	113.77 (17)	C8—C9—C91	122.01 (17)
C8—C1—C2	123.16 (18)	C9—C10—C11	118.57 (17)
C1—C2—C3	115.23 (18)	C9—C10—C12	121.95 (17)
C1—C2—H2A	108.5	C11—C10—C12	119.48 (17)
C3—C2—H2A	108.5	N1—C11—O1	120.39 (17)
C1—C2—H2B	108.5	N1—C11—C10	123.46 (17)
C3—C2—H2B	108.5	O1—C11—C10	116.15 (16)
H2A—C2—H2B	107.5	N2—C12—C10	178.3 (3)
C4—C3—C2	117.25 (19)	O1—C13—H13A	109.5
C4—C3—H3A	108.0	O1—C13—H13B	109.5
C2—C3—H3A	108.0	H13A—C13—H13B	109.5
C4—C3—H3B	108.0	O1—C13—H13C	109.5
C2—C3—H3B	108.0	H13A—C13—H13C	109.5
H3A—C3—H3B	107.2	H13B—C13—H13C	109.5
C5—C4—C3	116.56 (19)	C96—C91—C92	119.9 (2)
C5—C4—H4A	108.2	C96—C91—C9	120.6 (2)
C3—C4—H4A	108.2	C92—C91—C9	119.5 (2)
C5—C4—H4B	108.2	C93—C92—C91	120.0 (3)
C3—C4—H4B	108.2	C93—C92—H92	120.0
H4A—C4—H4B	107.3	C91—C92—H92	120.0
C6—C5—C4	116.2 (2)	C94—C93—C92	120.0 (4)
C6—C5—H5A	108.2	C94—C93—H93	120.0
C4—C5—H5A	108.2	C92—C93—H93	120.0
C6—C5—H5B	108.2	C93—C94—C95	120.8 (3)
C4—C5—H5B	108.2	C93—C94—H94	119.6
H5A—C5—H5B	107.4	C95—C94—H94	119.6
C5—C6—C7	115.56 (19)	C94—C95—C96	119.0 (4)
C5—C6—H6A	108.4	C94—C95—H95	120.5
C7—C6—H6A	108.4	C96—C95—H95	120.5
C5—C6—H6B	108.4	O2—C96—C91	115.1 (2)
C7—C6—H6B	108.4	O2—C96—C95	124.7 (3)
H6A—C6—H6B	107.5	C91—C96—C95	120.2 (3)
C8—C7—C6	113.47 (18)	O2—C97—H97A	109.5
C8—C7—H7A	108.9	O2—C97—H97B	109.5
C6—C7—H7A	108.9	H97A—C97—H97B	109.5
C8—C7—H7B	108.9	O2—C97—H97C	109.5
C6—C7—H7B	108.9	H97A—C97—H97C	109.5
H7A—C7—H7B	107.7	H97B—C97—H97C	109.5
C1—C8—C9	117.91 (17)	C11—N1—C1	118.11 (16)
C1—C8—C7	121.13 (17)	C11—O1—C13	117.98 (16)
C9—C8—C7	120.86 (17)	C96—O2—C97	118.4 (3)
C10—C9—C8	118.87 (17)		
			
N1—C1—C2—C3	96.0 (2)	C12—C10—C11—O1	1.8 (3)
C8—C1—C2—C3	−82.6 (3)	C10—C9—C91—C96	104.5 (2)
C1—C2—C3—C4	74.9 (3)	C8—C9—C91—C96	−78.0 (3)
C2—C3—C4—C5	−69.3 (3)	C10—C9—C91—C92	−75.5 (2)
C3—C4—C5—C6	99.4 (2)	C8—C9—C91—C92	102.0 (2)
C4—C5—C6—C7	−54.6 (3)	C96—C91—C92—C93	0.4 (3)
C5—C6—C7—C8	−51.8 (3)	C9—C91—C92—C93	−179.6 (2)
N1—C1—C8—C9	0.3 (3)	C91—C92—C93—C94	0.4 (5)
C2—C1—C8—C9	178.87 (18)	C92—C93—C94—C95	−0.4 (5)
N1—C1—C8—C7	−176.05 (18)	C93—C94—C95—C96	−0.4 (5)
C2—C1—C8—C7	2.5 (3)	C92—C91—C96—O2	178.5 (2)
C6—C7—C8—C1	90.8 (2)	C9—C91—C96—O2	−1.5 (3)
C6—C7—C8—C9	−85.4 (2)	C92—C91—C96—C95	−1.2 (3)
C1—C8—C9—C10	1.1 (3)	C9—C91—C96—C95	178.8 (2)
C7—C8—C9—C10	177.50 (18)	C94—C95—C96—O2	−178.5 (3)
C1—C8—C9—C91	−176.42 (18)	C94—C95—C96—C91	1.2 (4)
C7—C8—C9—C91	0.0 (3)	O1—C11—N1—C1	−179.78 (17)
C8—C9—C10—C11	−1.8 (3)	C10—C11—N1—C1	0.2 (3)
C91—C9—C10—C11	175.77 (18)	C8—C1—N1—C11	−1.0 (3)
C8—C9—C10—C12	177.52 (19)	C2—C1—N1—C11	−179.66 (17)
C91—C9—C10—C12	−4.9 (3)	N1—C11—O1—C13	−4.4 (3)
C9—C10—C11—N1	1.2 (3)	C10—C11—O1—C13	175.6 (2)
C12—C10—C11—N1	−178.16 (19)	C91—C96—O2—C97	177.0 (2)
C9—C10—C11—O1	−178.80 (16)	C95—C96—O2—C97	−3.3 (4)

### Hydrogen-bond geometry (Å, º)

*Cg*1 is the centroid of the pyridine ring.

**Table:**

*D*—H···*A*	*D*—H	H···*A*	*D*···*A*	*D*—H···*A*
C3—H3*A*···*Cg*1^i^	0.97	2.64	3.742 (2)	134

Symmetry code: (i) −*x*+1/2, *y*+1/2, −*z*+1/2.
